# Experimental Study of Rheological Behavior of MWCNT-Al_2_O_3_/SAE50 Hybrid Nanofluid to Provide the Best Nano-lubrication Conditions

**DOI:** 10.1186/s11671-021-03639-3

**Published:** 2022-01-04

**Authors:** Mohammad Hemmat Esfe, Soheyl Alidoust, Erfan Mohammadnejad Ardeshiri, Mohammad Hasan Kamyab, Davood Toghraie

**Affiliations:** 1 Department of Mechanical Engineering, Imam Hossein University, Tehran, Iran; 2 Department of Mechanical Engineering, Khomeinishahr Branch, Islamic Azad University, Khomeinishahr, Iran; 3 School of Chemistry, Damghan University, Damghan, 36716-41167 Iran

**Keywords:** Hybrid nano-lubricants, Rheological behavior, Numerical simulation, Nano-lubrication conditions

## Abstract

In this study, MWCNT-Al_2_O_3_ hybrid nanoparticles with a composition ratio of 50:50 in SAE50 base oil are used. This paper aims to describe the rheological behavior of hybrid nanofluid based on temperature, shear rate ($\dot{\gamma })$ and volume fraction of nanoparticles ($\varphi$) to present an experimental correlation model. Flowmetric methods confirm the non-Newtonian behavior of the hybrid nanofluid. The highest increase and decrease in viscosity (${\mu }_{\rm nf}$) in the studied conditions are measured as 24% and − 17%, respectively. To predict the experimental data, the five-point-three-variable model is used in the response surface methodology with a coefficient of determination of 0.9979. Margin deviation (MOD) of the data is determined to be within the permissible limit of − 4.66% < MOD < 5.25%. Sensitivity analysis shows that with a 10% increase in $\varphi$ at $\varphi =$ 1%, the highest increase in ${\mu }_{\rm nf}$ of 34.92% is obtained.

## Introduction

**Fig. 1:**
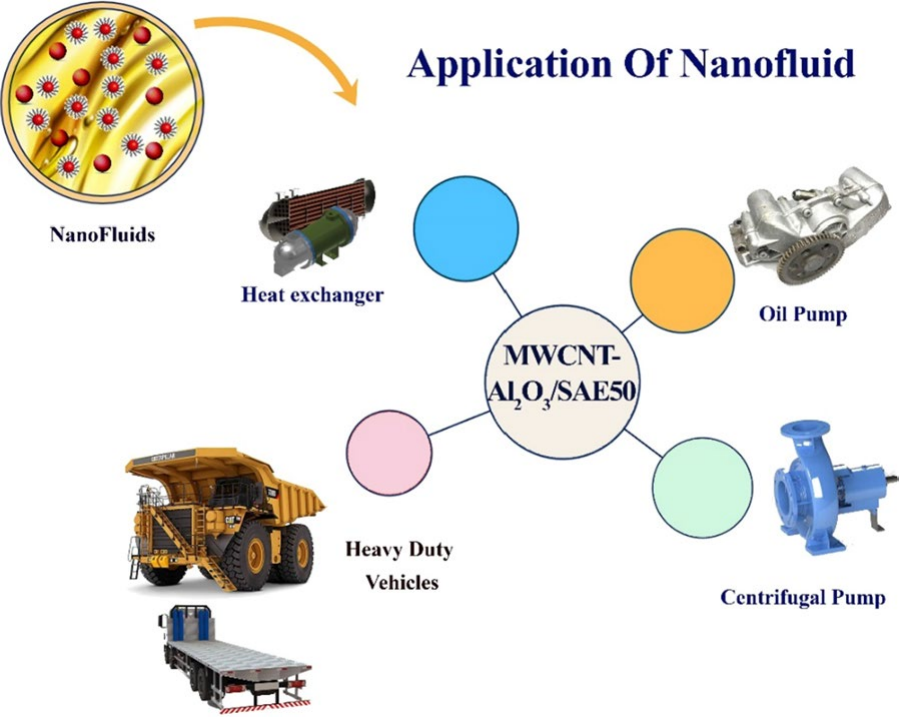
Application of nanofluids

Nano-sized particle suspensions in conventional liquids such as water, ethylene glycol, and oil are called nanofluids. Because of the high thermal conductivity and thermal performance of nanofluids compared to conventional liquids, it has attracted the attention of many researchers in recent years [1–3]. In 1995, Choi [4] introduced the term nanofluids to describe the applications of nanofluids extensively in various thermal systems such as heat exchangers, heating engines, and electronic devices (see Fig. 1). Undoubtedly, nanofluid viscosity (${\mu }_{\mathrm{nf}}$) and thermal conductivity are two important factors for nanofluids that have a direct effect on heat transfer and mass [5–7]. The addition of nanoparticles improves the thermal properties of nanofluids such as thermal conductivity and ${\mu }_{\mathrm{nf}}$, which is very important in various industries [6–8]. Although much scientific research was done in some industries, including the oil industry, to improve its quality, the research is still in the theoretical phase and researchers need to implement the laboratory results in practice [9, 10].

In recent years, the research team of Hemmat Esfe[11] started an active team in the field of nanofluid studies. The team also opened up new avenues in optimizing the properties of nanofluids. Esfe et al. [12] investigated the ${\mu }_{\mathrm{nf}}$ changes of MWCNT-Al_2_O_3_ nanoparticles with 5W50 base fluid at different temperatures and volume fraction of nanoparticles ($\varphi )$. The test results show that the ${\mu }_{\rm nf}$ increases with increasing $\varphi$. The reason for the increase in ${\mu }_{\rm nf}$ due to the increase in $\varphi$ is the effect of van der Waals force between molecules due to the formation of nano-clusters in the base fluid. Tian et al. [13] investigated the changes in viscosity and thermal conductivity of the base fluid after the addition of MWCNT-Al_2_O_3_ nanoparticles at *T* = 25 to *T* = 65 °C at different $\varphi$. The results of this study show that with increasing *T*, the ${\mu }_{\rm nf}$ decreases and heat transfer increases. Esfe et al.[14] investigated the rheological behavior of MWCNT-TiO_2_/SAE50 hybrid nanofluid with $\varphi$=0% to 1% at *T* = 25 and *T* = 50 °C and different $\dot{\gamma }$. The experimental results show that the nanofluid behavior in the relationship between shear stress and $\dot{\gamma }$ at all $\varphi$ is non-Newtonian. Chen et al. [15] investigated changes in the viscosity of the base oil after the addition of MWCNTs-TiO_2_ nanoparticles to the SAE50 base fluid. The results of these two experimental methods show that ANN is more reliable than curve fitting. In another study, Jilin et al. [16] investigated the effect of temperature and $\varphi$ on the ${\mu }_{\rm nf}$ of SAE50 engine oil in the presence of ZnO nanoparticles. According to the results of the reported experiments at *T* = 25 to 65 °C and different $\varphi$, the ${\mu }_{\rm nf}$ increases with increasing $\varphi$ up to 25.3% compared to the base oil. Asadi et al. [16]. investigated the changes in rheological behavior and ${\mu }_{\rm nf}$ of MWCNT/MgO hybrid nanofluid in SAE50 engine oil. These experiments were performed at $\varphi$=0.25% to 2% and a temperature of *T* = 25 to 50 °C. The experimental results show that the nanofluid behavior at all temperatures and $\varphi$ is Newtonian. In addition, experimental results show that increasing $\varphi$ leads to an increase in the ${\mu }_{\rm nf}$ at all temperatures. But with increasing temperature, the ${\mu }_{\rm nf}$ decreases. It was also observed that the maximum increase in ${\mu }_{\rm nf}$ at $\varphi =$ 2% and *T* = 40 °C was + 65%, while the lowest increase at $\varphi =$ 0.25% and *T* = 25 °C was 14.4%.

In a study, the effect of $\varphi$ and temperature on the behavior of nanofluid rheology with Water-EG/Al_2_O_3_ formulation with different composition ratios was investigated. Their laboratory observations show that the maximum increase in viscosity of nano-lubricants at *T* = 0 °C and $\varphi$=1.5% is equal to 2.58% [17]. In another study, the behavior of nanofluid rheology with Al_2_O_3_/water formulation was studied. The aim of the researchers in this paper is to investigate the effect of effective factors of temperature and $\varphi$ on viscosity. Their laboratory findings show that with increasing the $\varphi$ up to 5%, the maximum viscosity is 135% [18]. In 2020, a study was performed on Al_2_O_3_/ZnO-water nanofluid to investigate the viscosity of the nanofluid. Their laboratory observations show that at a $\varphi$ =1.67% and *T* = 25 °C, the maximum increase in viscosity was 96.37% [19]

**Fig. 2:**
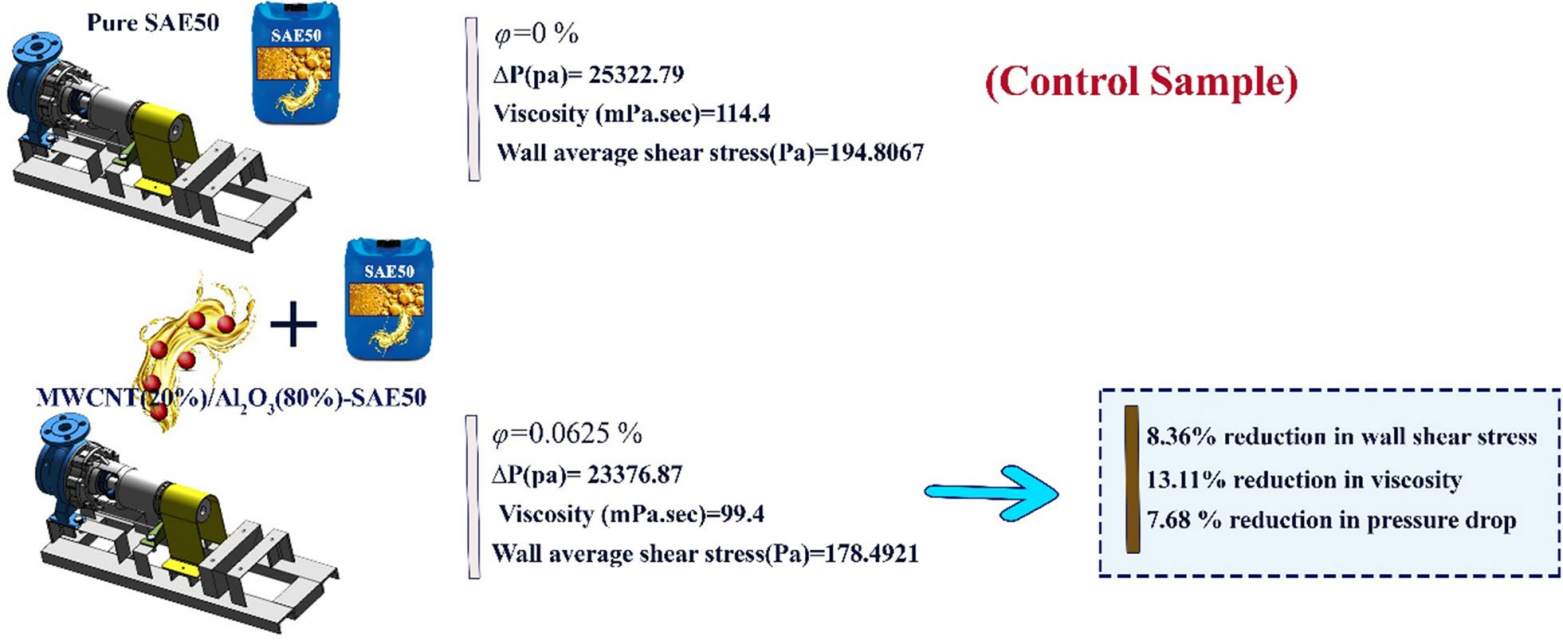
The performance of nano-lubricants at different conditions

This research, it was tried to take a comprehensive look at all aspects of nano-lubricant flow in the base fluid. The approach of the paper is to provide a comprehensive report on the performance of nano-lubricants at different conditions by analyzing the rheological behaviors of nano-lubricant (see Fig. 2). According to the laboratory data for nano-lubricants, the performance of ${\mu }_{\rm nf}$ and the extent of its effect from independent variables was plotted graphically and the best nano-lubricants suitable for different operating conditions in $\varphi$ and temperature based on the results extracted from analytical methods. In this research, nano-lubricants will be compared in separate sections with different purposes. In the first part of this study, the type of nano-lubricants (Newtonian and non-Newtonian) is studied and classified by the proposed methods. In the middle section, the role of nano-lubricant quality in increasing the life of components and upgrading is discussed. Then, the slope of the graph (${\mu }_{\rm nf}$-Temperature) was examined and calculated to determine the optimal viscosity index as one of the influential factors in evaluating the quality of nano-lubricants. Also, the optimal ${\mu }_{\rm nf}$ was modeled and investigated using the RSM. The error values of the predicted values with laboratory values were calculated and reported using the MOD method. Finally, the calculation of the thermal performance index for different states shows that the use of nano-lubricants and helical coils instead of the base fluid and Straight tubes improves the flow performance, ${\mu }_{\rm nf}$ and heat transfer.

## Methods/Experiment

### Characterizations

**Fig. 3:**
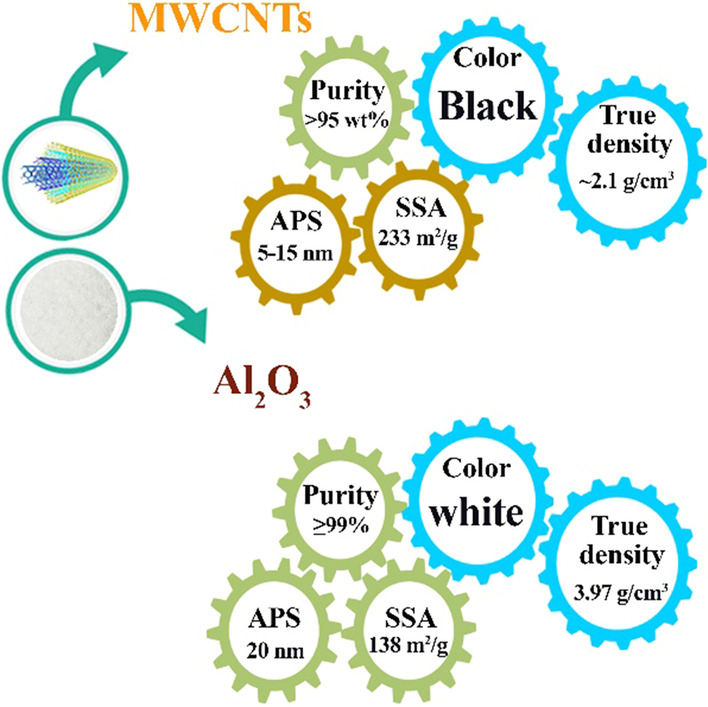
Physical and surface characteristics of applied nanoparticles

Al_2_O_3_ and MWCNT nanoparticles were used for injection in SAE50 base oil with a 50:50 ratio with MWCNT-Al_2_O_3_/SAE50 formulation. The used nanoparticles are US-nano research products, which are graphically reported in Fig. 3 of the physical properties of the studied nanoparticles.

Equation 1 was used to prepare hybrid nano-lubricants at different $\varphi$. Based on Eq. 1, the required mass percentage of each nanoparticle for each of the different $\varphi$ can be calculated and weighed using a compact digital balance device (no air weight interference) with an accuracy of 0.001 g. In Eq. 1, w is the weight of the nanoparticles, ρ is the density of the nanoparticles and φ is the volume fraction of the nanoparticles.

1 $$\mathrm{\varphi }= \frac{{\left.\frac{w}{\rho }\right|}_{\mathrm{MWCNT}}+{\left.\frac{w}{\rho }\right|}_{{\mathrm{Al}}_{2}{\mathrm{O}}_{3}}}{{\left.\frac{w}{\rho }\right|}_{\mathrm{MWCNT}}+{\left.\frac{w}{\rho }\right|}_{{\mathrm{Al}}_{2}{\mathrm{O}}_{3}}+{\left.\frac{w}{\rho }\right|}_{\mathrm{SAE}50}}\times 100$$

**Fig. 4:**
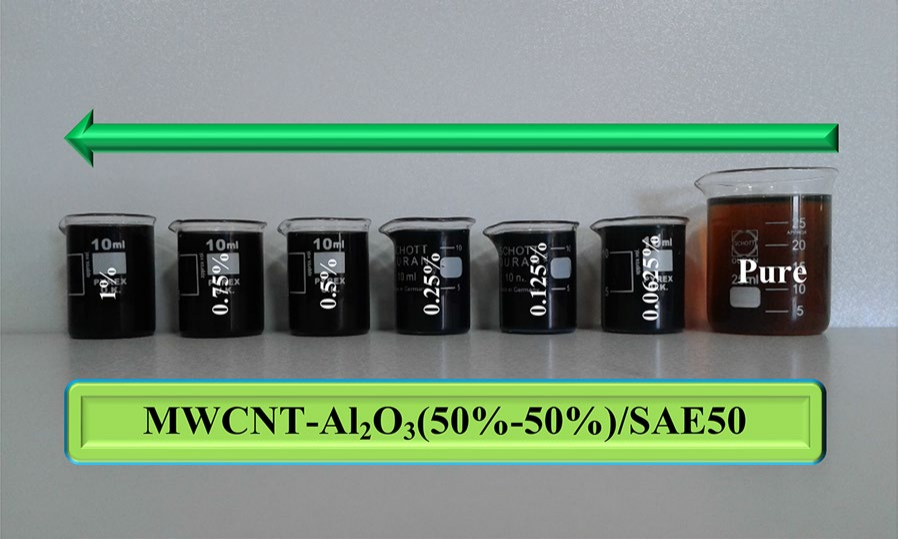
Stability of non-lubricants at different *φ*

To homogenize the nanoparticles in a certain combination within the base oil, a magnetic stirrer was used for 1 h. Using a magnetic stirrer, the nanosuspension was created with good stability. To increase the quality and reduce the instability of the nano-lubricant, an ultrasonic device was used for 1 h, resulting in no sedimentation, as well as breaking of the nanoparticle clusters. Figure 4 shows the stability of nanofluids in different volume fractions from 0 to 1% for three weeks. During this period, visual observations have shown that no sedimentation has occurred.

A laboratory rotary viscometer was used to measure the ${\mu }_{\mathrm{nf}}$. The Brookfield viscometer model CAP2000 + was used to measure the ${\mu }_{\mathrm{nf}}$. The technical specifications of the viscometer are given in Table 1.

**Table 1:** Technical specifications of viscometer device

Specification	CAP 2000 +
Inlet Voltage	115–230 V
Inlet Frequency	50–60 Hz
Power consumption	Less than 345 V
Torque range	18,100 rpm
Speed	5–1000 rpm
Temperature	5–55 ˚C
Material	Conical spindles and thermal plates are made from tungsten carbide and the sample holder is made from Teflon
Impact of environmental factors	CAP 2000 + Viscometer needs to work in bellow conditions: Environmental temperature: 5–20 ˚C Humidity: 20–80%

### Measurement of ${\mu }_{\rm nf}$

Temperature, $\dot{\gamma }$ and $\varphi$ were introduced as input of the device, based on which ${\mu }_{\rm nf}$ was measured in about 174 different experiments. The range of measuring conditions of the device is listed in Table 2. To avoid test error and measurement accuracy, the calibration process was performed using a glycerin sample. To increase accuracy and reduce error, ${\mu }_{\rm nf}$ measurements were repeated twice in different laboratory conditions and then their mean was recorded. Some of the measured data are reported in Table 3.

**Table 2:** Range of conditions for measuring the ${\mu }_{\rm nf}$

Nano-lubricant	Range of laboratory conditions		
	*T* (°C)	$\varphi$(%)	$\dot{\gamma }$(s^−1^)
MWCNT-Al_2_O_3_(50%-50%)/SAE50	25–50	0.0625–1	666.5–7998

**Table 3:** Some data measured by a CAP2000 + viscometer

Nano-lubricant	$\varphi$ (%)	*T* (°C)	$\dot{\gamma }$(s^−1^)	$\mu$ (mPa s)
MWCNT-Al_2_O_3_(50%-50%)/SAE50	0.0625	25	1333	435
	0.125	30	2666	361.9
	0.25	35	3999	266.2
	0.5	40	5332	203.4
	0.75	45	6665	156.4
	1	50	7998	125.6

## Results and Discussion

### Structure and Surface Properties of Materials

**Fig. 5:**
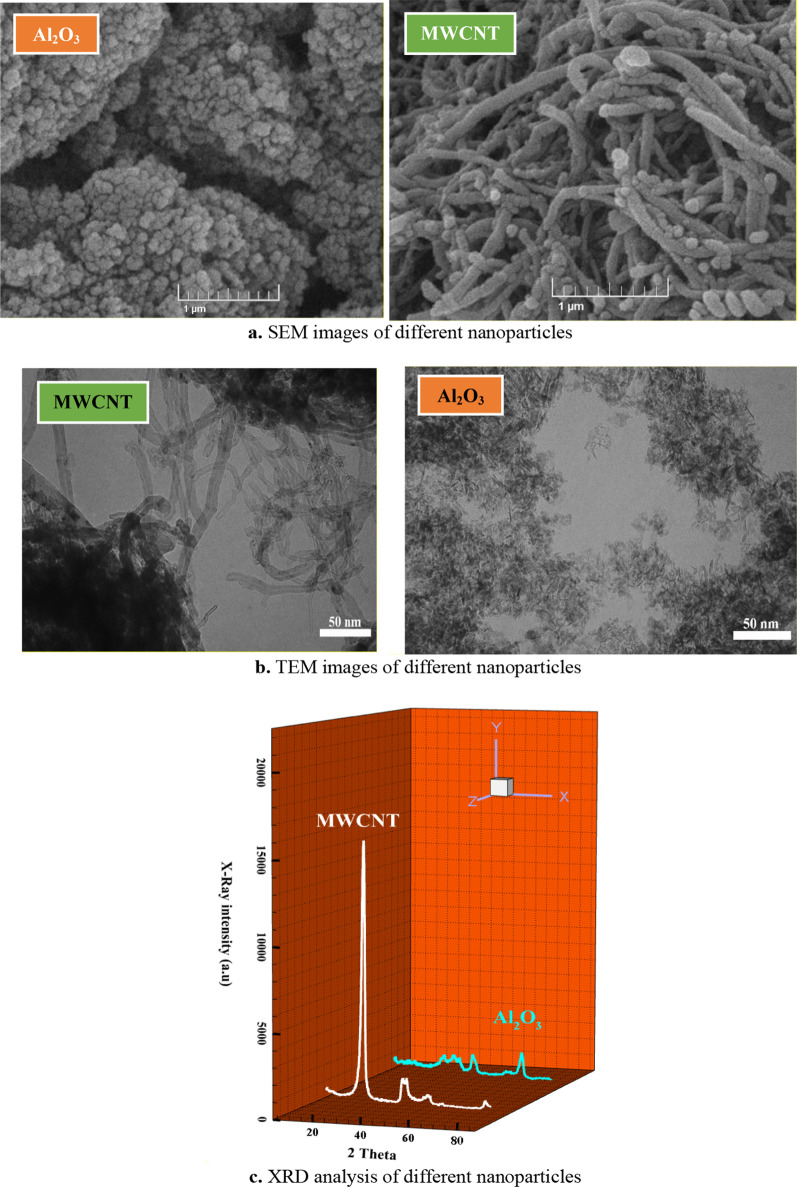
Images related to the use of SEM and TEM methods and XRD analysis

Today, advanced SEM and TEM imaging and X-ray diffraction (XRD) techniques are used to understand structure and surface properties of materials, as well as to determine their morphology (shape and size) [23–27]. According to Figure 5, SEM imaging was performed at a 1 μm scale and TEM at a 50 μm scale. Figure 5 shows the images related to the use of SEM and TEM methods and XRD analysis for the studied nanoparticles.

### Rheological Behavior

#### Effect of $\dot{\gamma }$

One method of analyzing the rheological behavior of nanofluids is to investigate the relationship between shear stress and shear rate applied to the fluid. According to Eq. 2, the slope of the equation is equal to the dynamic viscosity. Therefore, it is possible to determine the classification of fluids by determining the slope of the diagram. Based on the rheological behavior of liquids, they are classified into two main categories: Newtonian and non-Newtonian nanofluids. For Newtonian nanofluids, the viscosity remains constant for shear rate changes, while for non-Newtonian nanofluids, the viscosity becomes linear with changes in shear rate and shear stress [20].

2 $$\uptau =\upmu \frac{\mathrm{d}u}{\mathrm{d}y}=\mu \dot{\gamma }$$

**Fig. 6:**
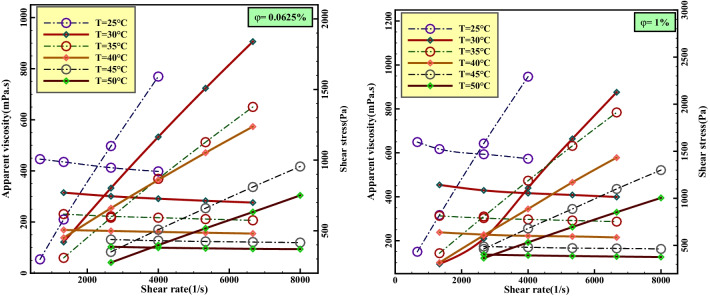
Shear stress and *μ*_nf_ change curves versus *γ* at different temperatures and *φ*

For accuracy and quality of correct detection of nanofluid behavior, two curves of Apparent viscosity-Shear rate (with negative and descending slope) and Shear stress-Shear rate (with ascending and positive slope) were used. The curves are plotted at the highest and lowest volume fractions under laboratory conditions and at *T* = 25–50 °C. Considering that the viscosity of the nanofluid in the Apparent viscosity-Shear rate diagram is variable for the changes in the shear rate, it can be concluded that the nanofluid is non-Newtonian. In other words, in pseudo-plastic fluids, their viscosity decreases when a force is applied, and the higher the force applied, the smoother the fluid, which is seen in Fig. 6. Also, considering the slope of the viscosity variable in the Shear stress-Shear rate diagram, it is another sign of confirmation of the non-Newtonian behavior of the nanofluid.

#### Power-Law Index

Alternatively, the power-law model is used to detect the rheological behavior of hybrid nano-lubricants to ensure that the behavior of the nano-lubricants is Newtonian and non-Newtonian. According to Eq. 3, the values of n refer to the flow index and determine the type of nano-lubricant behavior.

3 $$\uptau =m{\dot{\gamma }}^{n}$$

**Fig. 7:**
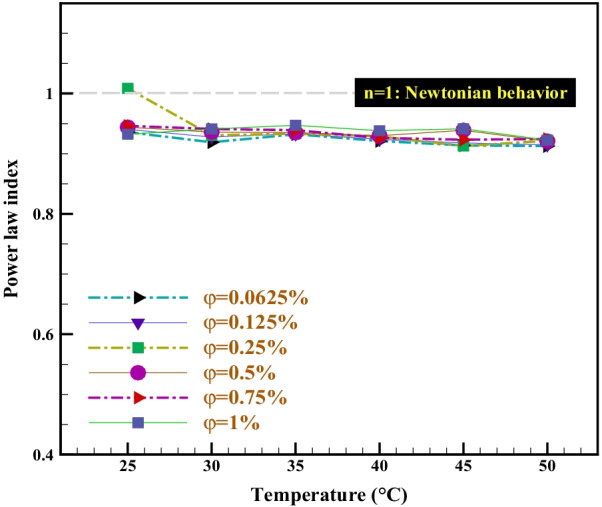
Effect of temperature and φ on power-law index

In these equations, m and n are two experimental parameters of curve fitting and are known as the coefficient of strength and flow behavior index, respectively. According to Eq. 3, for *n* > 1 the non-Newtonian behavior is dilatant, for *n* = 1 the behavior is the Newtonian, and for *n* < 1 the non-Newtonian behavior is pseudo-plastic. The results in Fig. 7 and Table 4 show that in all laboratory conditions, the values are *n* ≠ 1. Therefore, it can be concluded that nan-lubricant has a non-Newtonian behavior. One of the notable points in the diagram of Fig. 7 is the non-Newtonian behavior of a dilatant type (*n* = 1.0086) in $\varphi$ =0.25% and *T* = 25 °C, which is different from other studied conditions. By applying shear force, its viscosity increases.

**Table 4:** Power-law index values at different $\varphi$ and T

Nano-lubricants	Power-law index (*n*)						
		*T* = 25 °C	*T* = 30 °C	*T* = 35 °C	*T* = 40 °C	*T* = 45 °C	*T* = 50 °C
MWCNT-Al_2_O_3_ (50%-50%)/SAE50	$\mathrm{\varphi }$= 0.0625%	0.936	0.919	0.9323	0.9211	0.9139	0.9127
	$\mathrm{\varphi }$= 0.125%	0.9405	0.9277	0.9331	0.9238	0.9179	0.9148
	$\mathrm{\varphi }$= 0.25%	1.0086	0.9306	0.9333	0.9289	0.9129	0.9216
	$\mathrm{\varphi }$= 0.5%	0.9441	0.936	0.935	0.9299	0.939	0.9209
	$\mathrm{\varphi }$= 0.75%	0.9462	0.9411	0.9392	0.9263	0.9233	0.9245
	$\mathrm{\varphi }$= 1%	0.9324	0.9411	0.9472	0.9382	0.9414	0.9225

### Viscosity Comparison

#### Relative Viscosity

**Fig. 8:**
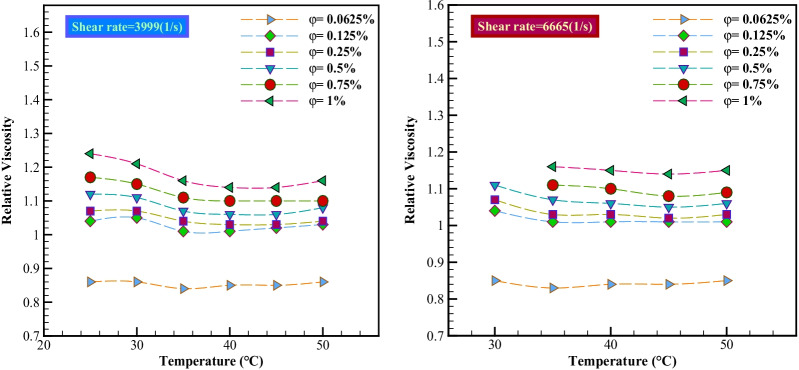
Relative viscosity in terms of φ at different temperatures and *γ* = 3999 and 6665 s^−1^

By dividing the ${\mu }_{\mathrm{nf}}$ per viscosity of base oil, a new concept called relative viscosity is derived. The relative viscosities of the studied nano-lubricants are calculated by Eq. 4 and examined in terms of temperature changes in Fig. 8. Relative viscosity values greater than 1 indicate an increase in the ${\mu }_{\mathrm{nf}}$ relative to the base fluid, and conversely, values less than 1 indicate a decrease in the ${\mu }_{\rm nf}$ relative to its base fluid.

4 $$\mathrm{Relative \ viscosity }\left(\mathrm{\%}\right)=\frac{{\mu }_{\rm nf}-{\mu }_{bf}}{{\mu }_{bf}}\times 100$$

According to Fig. 8, the relative viscosity for nano-lubricants is above line 1 in most $\varphi$, but a decrease in viscosity at $\varphi$ =0.0625% is observed at all temperatures. At low $\varphi$ due to the presence of fewer nanoparticles, the slip of the nano-lubricant layer compared to each other has occurred and this is one of the causes of the decrease in viscosity of the nanoparticles, the slip of the nano-lubricant relative to the base oil. However, at high $\varphi$, due to the presence of too many nanoparticles, it increases the sliding resistance between the nanoparticles, the slip of the nano-lubricant layers, which may increase the viscosity of the nanoparticles relative to the base oil.

According to the results reported in Table 5, the highest viscosity loss was observed at $\varphi =$ 0.0625% and *T* = 35 °C (− 17%).

**Table 5:** Statistical data on the relative viscosity of nano-lubricants

Nano-lubricant	$\dot{\gamma }$(s^−1^)	*T* (°C)	$\frac{{\mu }_{\rm nf}-{\mu }_{bf}}{{\mu }_{bf}}\times 100$(%)			
			$\varphi =0.0625\mathrm{\%}$	$\varphi =0.125\mathrm{\%}$	$\varphi =0.75\mathrm{\%}$	$\varphi =1\mathrm{\%}$
MWCNT-Al_2_O_3_ (50%-50%)/SAE50	3999 (300 rpm)	25	0.86	1.04	1.17	1.24
		30	0.86	1.05	1.15	1.21
		35	0.84	1.01	1.11	1.16
		40	0.85	1.01	1.10	1.14
	6665 (500 rpm)	30	0.85	1.04	–	–
		35	0.83 (− 17%)	1.01	1.11	1.16
		40	0.84	1.01	1.10	1.15
		45	0.84	1.01	1.08	1.14
		50	0.85	1.01	1.09	1.15

#### The Effect of Temperature on ${\mu }_{\rm nf}$

**Fig. 9:**
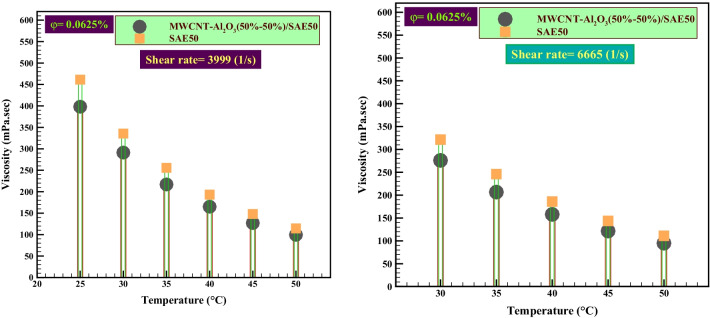
Comparison of the effect of temperature on *μ*_nf_ at *φ* = 0.0625%

In the last part of this study, the ${\mu }_{\rm nf}$-temperature curves, which express the effect of temperature and $\varphi$ of the experiment after the addition of nanoparticles in the base oil, were evaluated. In Fig. 9, the ${\mu }_{\rm nf}$ relative to the base fluid based on the temperature at the highest and lowest $\dot{\gamma }$ of 3999 s^−1^ and 6665 s^−1^, with the lowest $\varphi$ (0.0625%), is investigated. Figure 9 shows the reduction in ${\mu }_{\rm nf}$ at all test temperatures. The results of the statistical study in Tables 6 and 7 can accurately show the exact value of the difference between the viscosity of the nano-lubricant and the base fluid. One of the most important results is that the dynamic viscosity of a fluid is a function of temperature. In fact, all laboratory data point to the fact that the viscosity of nanofluids is a strong function of temperature and a weak function of pressure. Because nanofluids are incompressible materials, it is expected that the functional form of the viscosity of nanofluids can exhibit similar functional behavior.

**Table 6:** A comparative study of the effect of temperature on the ${\mu }_{\rm nf}$ relative to the base fluid

$\dot{\gamma }$(s^−1^)	*T* (°C)	$\Delta {\left({{\varvec{\mu}}}_{{\varvec{n}}-{\varvec{b}}}\right)}_{{\varvec{f}}}$(mPa s)
		MWCNT-Al2O3(50%-50%)/SAE50
3999	25	− 63.10
	30	− 44.40
	35	− 38.70
	40	− 28.10
6665	35	− 39.40
	40	− 28.10
	45	− 22.10
	50	− 16.10

Also, for the accurate and statistical study of the ${\mu }_{\rm nf}$ behavior of hybrid nano-lubricants, the difference between the viscosities of nano-lubricants and the base fluid at unique temperatures and $\varphi$ (0.0625%) was calculated and is reported in Table 6. The results of Table 7 show that nano-lubricants have a high viscosity drop compared to the base oil in all temperature ranges and  $\varphi$ = 0.125%. This nano-lubricant at *T* = 25 °C had the highest difference of − 63.10 mPa s (− 13.68%) with the base fluid. The results of these nano-lubricants at high temperatures are slightly different from the viscosity of the SAE50 base fluid.

**Table 7:** A comparative study of the effect of temperature on the viscosity relative to the base fluid

$\dot{\gamma }$ (s^−1^)	*T* (°C)	$\Delta {\left({{\varvec{\mu}}}_{{\varvec{n}}-{\varvec{b}}}\right)}_{{\varvec{f}}}$(mPa s)
		MWCNT-Al_2_O_3_ (50%–50%)/SAE50
3999	25	20.10
	30	16.90
	35	5.00
	40	3.80
6665	35	4.10
	40	3.00
	45	1.90
	50	1.90

**Fig. 10:**
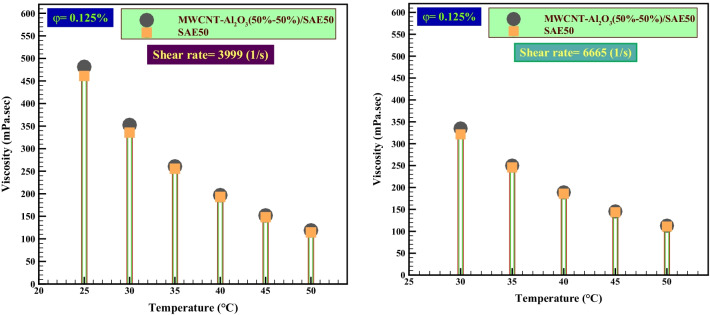
Comparison of the effect of temperature on ${\mu }_{\rm nf}$ at $\varphi$=0.125%

Figure 10 also compares the effect of temperature on the viscosity of nano-lubricant and base oil at $\varphi$=0.125% and 0.1%. However, no decrease in ${\mu }_{\rm nf}$ was observed in the fraction of higher $\varphi$.

The results of the review and comparison of Table 7 confirm the claims made in the analysis of Fig. 10. Nano-lubricants did not have a decrease in ${\mu }_{\rm nf}$ compared to the base oil at a higher $\varphi$.

## Comparison of Present Laboratory Results with Similar Researches

**Fig. 11:**
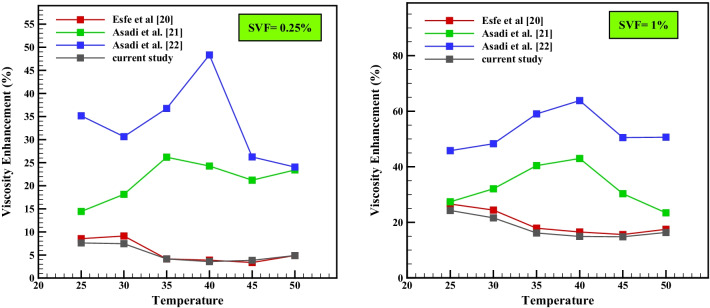
Comparison of different studies with the present study

In this section, an attempt was made to investigate the rheological behavior of nano-lubricants compared to some similar studies in a comparative manner in Fig. 11 under corresponding and equal conditions in $\varphi$ =0.25% and 1% and *T* = 25–50 °C. According to the comparison made in Table 8, it can be seen that at a high volume fraction equal to 1%, the present study has experienced less viscosity increase than most similar studies, so that the highest viscosity increase was + 24.26%. Also, in $\varphi$ and low temperature equal to 0.25% and *T* = 25 °C, it had the lowest increase in viscosity compared to other studies. In other words, at low concentrations compared to other similar nanofluids, there was a greater decrease in viscosity, so that the lowest increase in viscosity was equal to + 3.57%. Therefore, the studied nanofluid has shown better rheological behavior at different volume concentrations and can provide higher efficiency in the application of industry.

**Table 8:** Statistical comparison between the present study and similar research based on Fig. 11

SVF (%)	*T* (°C)	Viscosity enhancement (%)			
		Esfe et al. [20]	Asadi et al. [21]	Asadi et al. [22]	Current study
0.25	25	8.54	14.43	35.14	7.61
	30	9.11	18.14	30.63	7.44
	35	4.14	26.18	36.74	4.14
	40	3.88	24.27	48.33	3.57
	45	3.37	21.21	26.23	3.84
	50	4.89	23.41	24.05	4.89
1	25	26.58	27.38	45.8	24.26
	30	24.4	32.06	48.29	21.6
	35	17.87	40.42	59.03	16.15
	40	16.51	42.97	63.83	14.91
	45	15.59	30.3	50.49	14.78
	50	17.48	23.41	50.63	16.34

## Impractical Results

### RSM Method

The RSM is a combination of mathematical and statistical methods, which is useful for fitting models and analyzing problems in which the independent parameters control the dependent parameters. The RSM is used to optimize the process parameters and identify optimal conditions by determining how the dependent variable relates to the independent variable. Experimental design software (DOE) was used to optimize the formulation obtained from RSM. According to the RSM, to construct the regression model, the analysis of the fifth-order model with a coefficient of determination of 0.9979 was used.

#### New Correlation

**Fig. 12:**
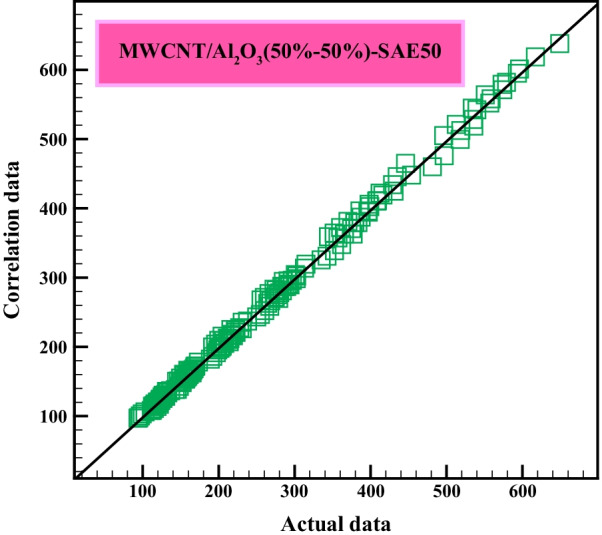
Correlation of predicted data with actual data

Equation 5 calculates the ${\mu }_{\rm nf}$ at *T* = 25–50 °C and $\varphi$=0.0625% to 1% and the $\dot{\gamma }$=6665 s^−1^ to 7998 s^−1^. The value *R*_sqr_ for mathematical Eq. 5 was set at 0.9979, which is satisfactory, as well shown in Fig. 12. It is worth noting that Eq. 5 can be used only within the scope of the studied conditions. Due to the presence of the shear rate factor in the relationship and its effect on the objective response function, the non-Newtonian behavior claimed in the laboratory can be correctly confirmed.

5 $$\begin{aligned} \mu_{{{\text{nf}}}} & = + {1993}.{47}0{57} - {93}.{819}0{\text{5T}} - 0.0{283}0{1}\dot{\gamma } + {1}.{\text{55329 T}}^{{2}} + {2751}.{424}0{8} \varphi^{{2}} + {1}.0{\text{7551 T}}^{{2}} \varphi \\ & \quad + {2}.{8931}0{\text{E}} - 00{\text{5 T}}^{{2}} \dot{\gamma } - {211}.{2}0{\text{468 T}}\varphi^{{2}} - {9}.{\text{34782E}} - 00{\text{3 T}}^{{3}} - {8}.0{389}0{\text{E}} - 00{\text{3 T}}^{{3}} \varphi \\ & \quad - {3}.{\text{65573E}} - 00{\text{7 T}}^{{3}} \dot{\gamma } + {3}0{8}.{\text{69349 T}}\varphi^{{3}} - {6445}.{21198}\varphi^{{4}} + {2}.{\text{66864E}} - 0{14}\dot{\gamma }^{{4}} - {144}.{\text{72883T}}\varphi^{{4}} \\ & \quad - {5}.{\text{54866E}} - 0{\text{16 T}}\dot{\gamma }^{{4}} + {4492}.{358}0{5}\varphi^{{5}} \\ \end{aligned}$$

**Fig. 13:**
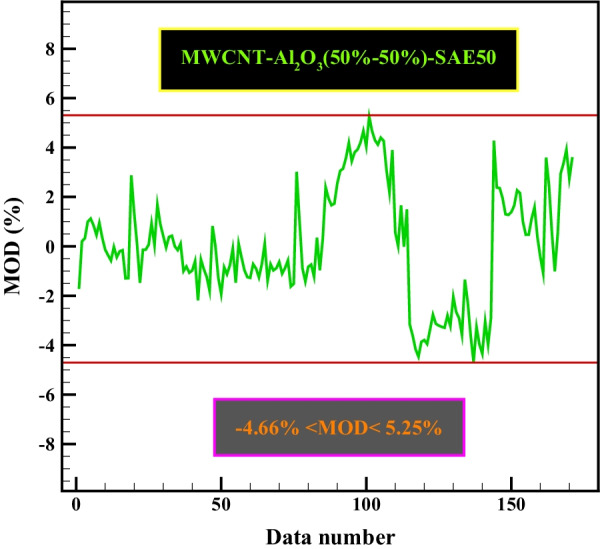
Range of MOD values in all laboratory data

Applications of the predictive mathematical model in this section include examining the correlation and agreement of the predicted data concerning the experimental data (Fig. 12), determining the MOD values (Fig. 13), and also examining the viscosity sensitivity to each factor affecting it was also mentioned. Figure 12 shows the complete consistency between the obtained figures from the mathematical equation and the laboratory results. As can be seen, in most cases, the experimental and correlation data overlap or show slight deviations. This behavior indicates that the proposed correlation has good accuracy. It can be inferred that the obtained mathematical relation has provided a suitable prediction model for estimating the ${\mu }_{\rm nf}$. Tables 9 and Table 10 provide statistical data related to the experimental model and effective parameters.

In Table 9, the value *R*^2^ indicates the fact that the percentage change of dependent variables in a problem is explained by the independent variable of the problem. In other words, the coefficient of determination or R^2^ indicates what the amounts of changes in the dependent variable of the problem is affected by the independent variable of the problem and to what extent the rest of the changes in the dependent variable of the problem are related to other factors in the problem. The coefficient of determination will always be between 0 and 100%, with the number 0 indicating that the model shows no correlation with the dependent and independent variables around its mean, and the number 100% indicating that the model shows all the variability of the response data. In Table 9, according to the coefficient of determination equal to 99.79%, it can be stated that the proposed experimental model with acceptable quality will predict the experimental data.

**Table 9:** Optimized modeling accuracy of three degrees

SD	6.68	R-Squared	0.9979
Mean	263.83	Adj R-Squared	0.9977
C.V. %	2.53	Pred R-Squared	0.9974
PRESS	8843.40	Adeq precision	258.970

**Table 10:** ANOVA-RSM fifth model

Source	Sum of squares	*df*	Mean square	*F* value	*p* value Prob > *F*	
Model	3.341E+006	16	2.088E+005	4678.37	< 0.0001	Significant
A-T	1.568E+005	1	1.568E+005	3512.35	< 0.0001	
C-Shear rate	4531.61	1	4531.61	101.52	< 0.0001	
A^2^	79,112.63	1	79,112.63	1772.25	< 0.0001	
B^2^	1961.02	1	1961.02	43.93	< 0.0001	
A^2^B	5480.95	1	5480.95	122.78	< 0.0001	
A^2^C	246.46	1	246.46	5.52	0.0200	
AB^2^	787.91	1	787.91	17.65	< 0.0001	
A^3^	3894.49	1	3894.49	87.24	< 0.0001	
A^3^B	438.98	1	438.98	9.83	0.0020	
A^3^C	1462.99	1	1462.99	32.77	< 0.0001	
AB^3^	4962.82	1	4962.82	111.18	< 0.0001	
B^4^	4199.91	1	4199.91	94.08	< 0.0001	
C^4^	1308.93	1	1308.93	29.32	< 0.0001	
AB^4^	1417.44	1	1417.44	31.75	< 0.0001	
AC^4^	319.98	1	319.98	7.17	0.0082	
B^5^	36,256.74	1	36,256.74	812.21	< 0.0001	
Residual	7008.42	157	44.64			
Cor total	3.348E+006	173				

#### Margin of Deviation (MOD)

One of the methods to verify the quality and accuracy of the experimental model is to use the MOD method [28, 29]. The MOD between the laboratory results and the experimental relationships extracted from Eq. 6 is as follows:

6 $${\rm MOD}=\frac{\mu_{rel_pre}{\mu_{rel_exp}}}{\mu_{rel_exp}} \times 100$$

Figure 13 shows the calculated MOD between the laboratory results and the experimental relationships at different temperatures and $\varphi$ based on Eq. 6. The maximum MOD was calculated to be 5.25%. Therefore, considering the maximum data in the appropriate range, the accuracy, quality, and validity of the model were acceptable.

#### Viscosity Sensitivity

Sensitivity analysis is the process of recognizing how changes in the outputs of a given model are due to changes in the input factors of the model (variables or parameters). For example, if a small change in input variables or model parameters results in a relatively large change in output, the output is said to be sensitive to variables or parameters. Sensitivity analysis is usually performed through a series of experiments in which the model maker uses different input values to determine how a change in input causes a change in the output of the model. Eq. (7) was used for sensitivity analysis.

7 $$Viscosity \ sensitivity =\frac{{\left({Viscosity}_{{ }_{after change}}\right)}_{Pre}-{\left({Viscosity}_{{ }_{before change}}\right)}_{Pre}}{{\left({Viscosity}_{{ }_{before change}}\right)}_{Pre}}\times 100$$

**Fig. 14:**
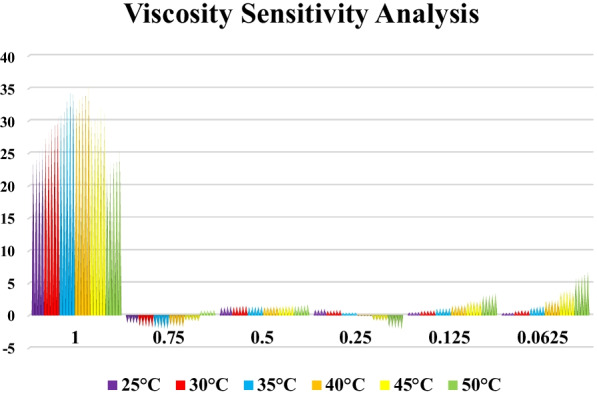
Viscosity sensitivity analysis for all data at different laboratory conditions

In Fig. 14, the values of viscosity sensitivity to $\varphi$ are plotted with + 10% variation. It was observed that at high $\varphi$ (1%), the highest sensitivity to changes was occurred, which is equal to 34.92%. Figure 14 shows that in variable volume fraction and constant temperature, a greater increase in sensitivity was occurred than in the case of variable temperature and constant volume fraction. Therefore, it can be concluded that the sensitivity of the objective function of the volume fraction was higher than temperature and the necessary considerations should be made in the preparation of nanofluids, especially in the volume fraction of 1% to reduce errors in rheological behavior analysis.

## Conclusion

In this study, an attempt was made to investigate for the first time the rheological behavior of hybrid nano-lubricants. Laboratory study of nano-lubricant behavior was performed based on temperature; $\dot{\gamma }$ and $\varphi$. Also, using RSM, a mathematical model was presented based on how the dependent variable is related to several independent variables. The results of the analysis are summarized as follows:

- Viscosity/$\dot{\gamma }$ and shear stress/$\dot{\gamma }$ diagrams show that at different laboratory conditions, the ${\mu }_{\rm nf}$ has a pseudo-plastic non-Newtonian behavior (*n* < 1).
- It was found that with increasing the nanoparticles in the base oil, the ${\mu }_{\rm nf}$ should increase so that at the highest $\varphi$ (1%), it has grown by 24%. Increasing the nanoparticles in the base fluid will increase the friction between the oil layers due to the collision of the nanoparticles with each other and increase the ${\mu }_{\rm nf}$ compared to base oil, which can be one of the main reasons for this.
- Laboratory findings show that at low $\varphi$ (0.0625%), due to the presence of nanoparticles in the oil layers and slipping between them, the ${\mu }_{\rm nf}$ decreases by 17%.
- The behavior of ${\mu }_{\rm nf}$ relationship to temperature, $\varphi$ and $\dot{\gamma }$ was observed as exponential-inverse function, multi-degree-direct function, and exponential-inverse function, respectively.
- The RSM has good accuracy and quality in predicting the experimental data so that the coefficient of determination and MOD are 0.9979 and − 4.66% < MOD < 5.25%, respectively.
- Sensitivity analysis shows that the highest ${\mu }_{\rm nf}$ sensitivity to $\varphi$=1% was occurred, which was equal to 34.92%, which requires the greatest care in the preparation of $\varphi$ by the laboratory operator.

## Data Availability

Not applicable.

## Abbreviations

RSM: Response surface methodology
MOD: Margin deviation
XRD: X-ray diffraction

## References

[1] Aberoumand S et al (2016) Experimental study on the rheological behavior of silver-heat transfer oil nanofluid and suggesting two empirical based correlations for thermal conductivity and viscosity of oil based nanofluids. Appl Therm Eng 101:362–372

[2] Li H et al (2015) Experimental investigation of thermal conductivity and viscosity of ethylene glycol based ZnO nanofluids. Appl Therm Eng 88:363–368

[3] Toghraie D et al (2019) Designing an Artificial Neural Network (ANN) to predict the viscosity of Silver/Ethylene glycol nanofluid at different temperatures and volume fraction of nanoparticles. Physica A 534:122142

[4] Wang Y et al (2021) Experimental analysis of hollow fiber membrane dehumidifier system with SiO_2_/CaCl_2_ aqueous desiccant solution. Energy Rep 7:2821–2835

[5] Soltani F et al (2020) Experimental measurements of thermal conductivity of engine oil-based hybrid and mono nanofluids with tungsten oxide (WO3) and MWCNTs inclusions. Powder Technol 371:37-44

[6] Saboori R et al (2017) Improvement of thermal conductivity properties of drilling fluid by CuO nanofluid. Transp Phenom Nano Micro Scales 5(2):97–101

[7] Hosseinian Naeini A et al (2016) Nanofluid thermal conductivity prediction model based on artificial neural network. Challenges Nano Micro Scale Sci Technol 4(2):41–46

[8] Alirezaie A et al (2018) Price-performance evaluation of thermal conductivity enhancement of nanofluids with different particle sizes. Appl Therm Eng 128:373–380

[9] Alirezaie A (2017). Investigation of rheological behavior of MWCNT (COOH-functionalized)/MgO-engine oil hybrid nanofluids and modelling the results with artificial neural networks. J Mol Liquids.

[10] Naddaf A, Heris SZ, Pouladi BJPT (2019) An experimental study on heat transfer performance and pressure drop of nanofluids using graphene and multi-walled carbon nanotubes based on diesel oil. Powder Technol 352:369–380

[11] Esfe MH et al (2019) Proposing new hybrid nano-engine oil for lubrication of internal combustion engines: Preventing cold start engine damages and saving energy. Energy 170:228–238

[12] Esfe MH (2017). Experimental investigation on non-Newtonian behavior of Al_2_O_3_-MWCNT/5W50 hybrid nano-lubricant affected by alterations of temperature, concentration and shear rate for engine applications. Int Commun Heat Mass Transfer.

[13] Tian X-X et al (2020) Efficacy of hybrid nano-powder presence on the thermal conductivity of the engine oil: an experimental study. Powder Technol 369:261–269

[14] Esfe MH (2019). Experimental investigation of effective parameters on MWCNT–TiO_2_/SAE50 hybrid nanofluid viscosity. J Therm Anal Calorim.

[15] Chen Z (2020). Applying artificial neural network and curve fitting method to predict the viscosity of SAE50/MWCNTs-TiO_2_ hybrid nanolubricant. Physica A: Stat Mech Appl.

[16] Ma J (2020). Viscosity, cloud point, freezing point and flash point of zinc oxide/SAE50 nanolubricant. J Mol Liquids.

[17] Sundar LS (2014). Thermal conductivity and viscosity of stabilized ethylene glycol and water mixture Al_2_O_3_ nanofluids for heat transfer applications: An experimental study. Int Commun Heat Mass Transfer.

[18] Chandrasekar M (2010). Experimental investigations and theoretical determination of thermal conductivity and viscosity of Al_2_O_3_/water nanofluid. Exp Therm Fluid Sci.

[19] Wole-Osho I (2020). An experimental investigation into the effect of particle mixture ratio on specific heat capacity and dynamic viscosity of Al_2_O_3_-ZnO hybrid nanofluids. Powder Technol.

[20] Esfe MH (2021). Experimental study of rheological characteristics of MWCNT-Al_2_O_3_ (40: 60)/SAE50 hybrid nano-lubricant to identify optimal lubrication conditions and post-processing of results using the response surface method. J Mater Res Technol.

[21] Asadi A et al (2016) The effect of temperature and solid concentration on dynamic viscosity of MWCNT/MgO(20:80)-SAE50 hybrid nano-lubricant and proposing a new correlation: an experimental study. Int Commun Heat Mass Trans 78:48–53

[22] Asadi A, Asadi M, Rezaniakolaei A, Rosendahl LA, Afrand M, Wongwises S (2018). Heat transfer efficiency of Al_2_O_3_-MWCNT/thermal oil hybrid nanofluid as a cooling fluid in thermal and energy management applications: an experimental and theoretical investigation. Int J Heat Mass Transf.

[23] Zhu H, Zhu J, Zhang Z, Zhao R (2021) Crossover from linear chains to a honeycomb network for the nucleation of hexagonal boron nitride grown on the Ni(111) surface. J Phys Chem C. 10.1021/acs.jpcc.1c09334

[24] Shen Z, Wang F, Wang Z, Li J (2021) A critical review of plant-based insulating fluids for transformer: 30-year development. Renew Sustainable Energy Rev 141:110783. 10.1016/j.rser.2021.110783

[25] Mu S, Liu Q, Kidkhunthod P, Zhou X, Wang W, Tang Y (2020) Molecular grafting towards high-fraction active nanodots implanted in N-doped carbon for sodium dual-ion batteries. Nat Sci Rev 8(7). 10.1093/nsr/nwaa178 10.1093/nsr/nwaa178 PMC8310755 34691681

[26] Cui X, Li C, Ding W, Chen Y, Mao C, Xu X, Sharmal S (2021) Minimum quantity lubrication machining of aeronautical materials using carbon group nanolubricant: from mechanisms to application. Chi J Aeronaut. 10.1016/j.cja.2021.08.011

[27] Sun J, Du H, Chen Z, et al (2021) MXene quantum dot within natural 3D watermelon peel matrix for biocompatible flexible sensing platform. Nano Res. 10.1007/s12274-021-3967-x

[28] Ruhani B, Abidi A, Kadhim Hussein A, Younis O, Degani M, Sharifpur M (2022) Numerical simulation of the effect of battery distance and inlet and outlet length on the cooling of cylindrical lithium-ion batteries and overall performance of thermal management system. J Energy Storage 45:103714

[29] Salehi M, Heidari P, Ruhani B, Kheradmand A, Purcar V, Căprărescu S (2021) Theoretical and experimental analysis of surface roughness and adhesion forces of MEMS surfaces using a novel method for making a compound sputtering target. Coatings 11(12):1551

